# Zinc-metal–organic frameworks with tunable UV diffuse-reflectance as sunscreens

**DOI:** 10.1186/s12951-022-01292-1

**Published:** 2022-02-19

**Authors:** Jisheng Xiao, Haishan Li, Wanling Zhao, Chengyuan Cai, Tingting You, Zhenyu Wang, Mengling Wang, Feng Zeng, Jinmei Cheng, Jiaxin Li, Xiaopin Duan

**Affiliations:** 1 Translational Medicine Research Center, Zhujiang Hospital, Southern Medical University/The Second School of Clinical Medicine, Southern Medical University, Guangzhou, 510515 Guangdong China; 2 Cancer Research Institute, School of Basic Medical Sciences, Southern Medical University, Guangzhou, 510515 China; 3 Science and Technology Innovation Center, Guangzhou University of Chinese Medicine, Guangzhou, 510405 China; 4 Department of Pharmacology, School of Pharmacy, Fudan University, Minhang Hospital, Shanghai, 201203 China; 5 Artemisinin Research Center, Institute of Science and Technology, The First Affiliated Hospital, The First Clinical Medical School, Lingnan Medical Research Center, Guangzhou University of Chinese Medicine, Guangzhou, 510405 China

**Keywords:** Sunscreens, Metal–organic frameworks, UV, Diffuse-reflectance, ZIF-8

## Abstract

**Background:**

UV exposure continues to induce many health issues, though commercial sunscreens are available. Novel UV filters with high safety and efficacy are urgently needed. Metal–organic frameworks (MOFs) could be a suitable platform for UV filter development, due to their tunable optical, electrical, and photoelectric properties by precise controlled synthesis.

**Results:**

Herein, four zinc-based MOFs with various bandgap energies were chose to investigate their optical behaviors and evaluate their possibility as sunscreens. Zeolitic imidazolate framework-8 (ZIF-8) was found to possess the highest and widest UV reflectance, thereby protecting against sunburn and DNA damage on mouse skin and even achieving a comparable or higher anti-UV efficacy relative to the commercially available UV filters, TiO_2_ or ZnO, on pig skin, a model that correlates well with human skin. Also, ZIF-8 exerted appealing characteristics for topical skin use with low radical production, low skin penetration, low toxicity, high transparency, and high stability.

**Conclusion:**

These results confirmed ZIF-8 could potentially be a safe and effective sunscreen surrogate for human, and MOFs could be a novel source to develop more effective and safe UV filters.

**Graphical Abstract:**

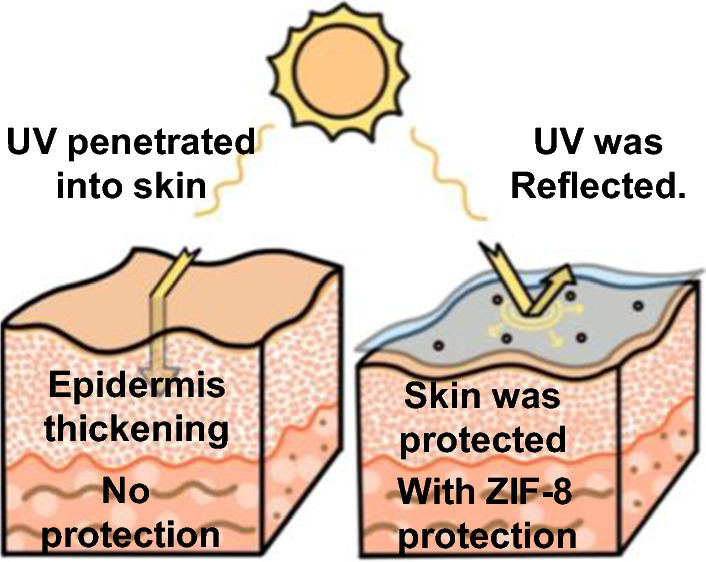

**Supplementary Information:**

The online version contains supplementary material available at 10.1186/s12951-022-01292-1.

## Introduction

Ultraviolet (UV) radiation produced by sun falls into three categories, including UVA at 400–320 nm, UVB at 320–280 nm, and UVC at 280–200 nm. UV plays a key role for the living things and regulates many biology processes. For example, UV radiation at a suitable dose increases the production of natural endorphins and Vitamin D in the skin. However, excessive exposure of UV would lead to health risks, such as atrophy, melanin deposition, aging and skin carcinogenesis [1]. UVA is reported to induce aging, immunosuppression, and skin carcinogenesis through generation of reactive oxygen species (ROS) or upregulation of immunosuppressive cytokines. UVB potentially causes sunburn, induces DNA damage through increasing the expression of cyclobutane pyrimidine dimers (CPDs), and even leads to non-melanoma skin cancers [2, 3].

Commercially available organic UV filters, including benzophenones, salicylates, octinoxates, etc., have been developed for anti-UV protection with the mechanisms to absorb UV radiation and release the energy through photoreactions, fluorescence, or energy redistribution within UV filter [4]. However, these organic molecules can pass through follicles or stratum corneum, penetrate into skin cells and induce skin complications [5]. Also, these organic compounds would be further absorbed and distributed into the whole body, which potentially results in systemic toxicities (e.g. endocrine disruption) [5]. Alternatively, inorganic sunblock agents, such as titanium dioxide (TiO_2_) and zinc oxide (ZnO) nanoparticles with UV absorption and scattering characteristics have been used to protect against UV exposure and address the transdermal penetration concern of organic UV filters. However, TiO_2_ can induce the generation of ROS with UV irradiation, which potentially lead to damage to cells and tissues, and even the development of skin cancer, though carcinogenesis of TiO_2_ is still controversial [6–8]. To address these concerns, current strategies, including decorating the nanoparticles with aluminum oxide or silica oxide or addition antioxidant molecules, have been developed to relieve the photocatalytic activity [6–8]. However, the results are not satisfied due to the degradation of antioxidants after exposure to the sun for a long time [9]. ZnO also is used as a surrogate for UV filter with no obvious local toxicity and no penetration of ZnO nanoparticles into viable epidermis [10, 11]. However, Zn content dissociated from ZnO was detected in blood [10], which would potentially cause unknown biological effects, though the reported Zn dose in circulation is relatively low. Moreover, TiO_2_ and ZnO suspensions are prone to be opaque, which is not favored for cosmetic reasons. Therefore, novel candidates with high anti-UV efficiency, photostability, physiological stability, and transparent characteristics in suspensions are urgently needed for the development of UV filters.

Metal–organic frameworks (MOFs), the hybrid polymers formed with metal nodes and organic ligands through coordination bonds, are predominantly used in gas absorption and separation, catalysis, sensing, and biomedicine due to the properties of high stability and porosity, large surface area, and tunable functionalities. Unfortunately, MOFs have not been well exploited for sunscreen development. Semiconductor MOFs (e.g. Zn, Mg, Cu, Ni, Mn, or Sr-based MOFs) [12] can transport photogenerated charges between metal centers or between the metal ions and linkages for long-distance by hydrogen-bonded vertical π stacks or full π-d conjugation [2, 12–17]. Specially, the bandgap of MOFs, which influences the maximal excitation wavelength (λ_max_), can be tuned by selecting various metal nodes and organic linkers, modifying the conjugation of the linkers, and functionalizing the linkers with other groups, etc. According to photoelectric effect Eq. (1), the light will be absorbed if the wavelength is lower than λ_max_ and scattered or reflected if the wavelength is higher than λ_max_.[12, 18] So, MOFs with tunable UV absorption and reflection characteristics due to structure variations provide us a novel channel to develop UV filters with on-demand anti-UV effects.

1 $$Eg = hv = h*\frac{c}{\lambda }\qquad \lambda = \frac{hc}{{Eg}}$$

where h is Planck constant (6.626 × 10^–34^ J·S), c is light velocity (3.0 × 10^8^ m s^−1^).

**Fig. 1:**
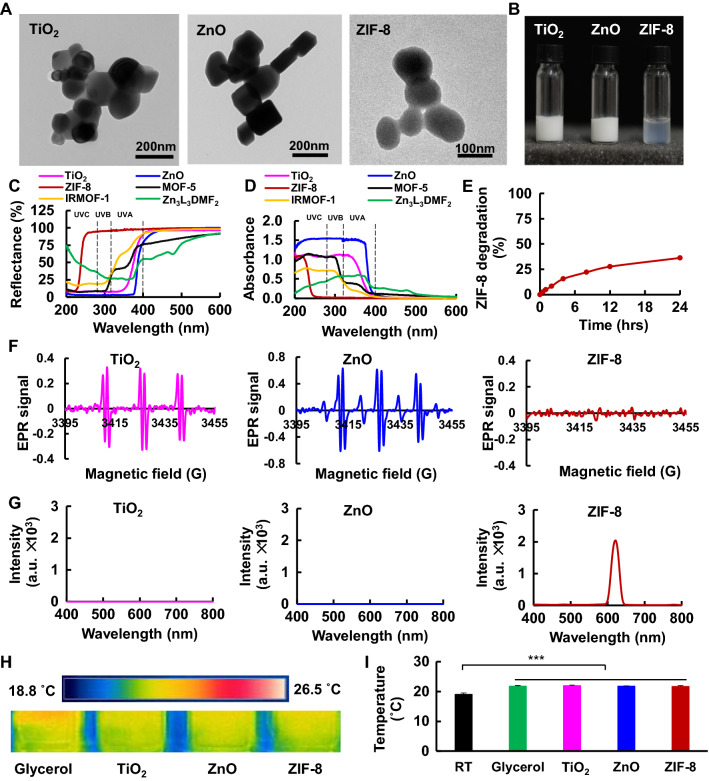
Physical and chemical characterizations of zinc-based MOFs. **A** TEM images of TiO_2_, ZnO and ZIF-8. **B** Digital photographs of TiO_2_, ZnO and ZIF-8. ZIF-8 suspension was more transparent relative to TiO_2_ and ZnO. **C** Diffuse reflection spectra and **D** calculated UV–Vis absorbance spectra for TiO_2_, ZnO and zinc-based MOFs. ZIF-8 showed the widest and highest UVA and UVB scattering with lowest λ_max_. **E** ZIF-8 degradation in artificial sweat (pH 6.5, 32 °C). **F** EPR spectra of POBN-OH spin abduct signal produced by TiO_2_, ZnO and ZIF-8 suspensions (800 µg mL^−1^ in ethanol). ZIF-8 induced less EPR signal compared to TiO_2_ after UV exposure. **G** Fluorescence spectra of TiO_2_, ZnO and ZIF-8 after excitation with UV light (308 nm). ZIF-8 showed an emission at 621 nm. **H** Thermographs and **I** quantitative analysis of glycerol, TiO_2_, ZnO and ZIF-8 after UV exposure for 2 h. (RT: room temperature) 2–3 °C temperature increases were observed for all four groups compared to room temperature

Herein, four zinc-based MOFs with different bandgap energies (Egs), including zeolitic imidazolate framework-8 (ZIF-8), Zn_3_L_3_DMF_2_, MOF-5 and IRMOF-1 (isoreticular MOF-5), were selected to study their optical characteristics and find the potential sunscreens. Among them, ZIF-8 showed the widest and highest scattering of UVA and UVB, followed by IRMOF-1, MOF-5, Zn_3_L_3_DMF_2_, TiO_2_ and ZnO (Fig. 1C, D, Additional file 1: Fig. S2), thus ZIF-8 was selected as the final model MOFs to assess its potential as UV filters. ZIF-8 was more transparent compared to ZnO and TiO_2_ with high physiological stability, low skin toxicity, and weak ROS production (Figs. 1B, E, F, 6A, B). On the mouse skin, ZIF-8 successfully inhibited epidermal hyperplasia and collagen degradation caused by UV exposure. On the Ba-Ma miniature pig skin, a model that correlates well with human skin, ZIF-8 achieved better effects to inhibit epidermal hyperplasia and DNA damage than ZnO, and comparable effect relative to TiO_2_. Furthermore, negligible ZIF-8 was found to penetrate mouse and pig skin, combined with low ROS generation, making it safer for clinical use. All these results confirmed ZIF-8 could potentially be used as a novel sunscreen surrogate for human. As we are investigating a new use of metal organic frameworks and how they perform on UV absorbance/reflectance with structure variations, we believe that our results are relevant to wide research areas including aesthetic medicine, pharmacy, and materials science.

## Results and discussion

### Physical and chemical characterizations of Zn-based MOFs

MOFs were successfully synthesized and the structures of MOFs, TiO_2_ and ZnO were confirmed in the experiment section (Additional file 1: Figs. S1–S16). TEM revealed sizes of 102.1 ± 18.9 nm and 114.8 ± 53.2 nm for TiO_2_ and ZnO, respectively, whereas ZIF-8 showed a similar size with 82.3 ± 24.5 nm in diameter (Fig. 1A, Additional file 1: Fig. S1), confirming TiO_2_, ZnO and ZIF-8 are in nanoparticulate form. The sizes for MOF-5, IRMOF-1, and Zn_3_L_3_DMF_2_ were 310.6 ± 142.4 nm, 47.1 ± 13.6 nm, and 256.0 ± 91.3 nm, respectively (Additional file 1: Fig. S13A–F). The zeta potentials also were measured, showing 31.7 ± 0.6 mV, 16.4 ± 0.7 mV, 29.5 ± 0.8 mV, − 9.9 ± 1.5 mV, − 7.0 ± 0.6 mV, and − 5.6 ± 0.6 mV for TiO_2_, ZnO, ZIF-8, MOF-5, IRMOF-1, and Zn_3_L_3_DMF_2_, respectively (Additional file 1: Fig. S13G). The previously reported bandgaps for MOFs, TiO_2_, and ZnO are ZIF-8 (5.5 eV) > IRMOF-1 (3.6 eV) > MOF-5 (3.4 eV) > TiO_2_ (3.2 eV) = ZnO (3.2 eV) > Zn_3_L_3_DMF_2_ (3.1 eV) [12, 19, 20], and the calculated λ_max_ order should be ZIF-8 < IRMOF-1 < MOF-5 < TiO_2_ = ZnO < Zn_3_L_3_DMF_2_ according to photoelectric effect Eq. (1). However, the diffuse-reflectance results showed ZIF-8 exerted lowest λ_max_ (236 nm), followed by IRMOF-1 (λ_max_: 290 nm), MOF-5 (λ_max_: 294 nm), TiO_2_ (λ_max_: 340 nm), Zn_3_L_3_DMF_2_ (λ_max_: 366 nm) and ZnO (λ_max_: 370 nm) (Fig. 1C, D, Additional file 1: Fig. S2), indicating the acquired λ_max_ order from experiment did not correlate well with the calculated one, which could be due to red and blue shifts caused by the quantum size effect and dielectric confinement effect [21]. As shown in the Eq. (2) for the lowest exciton energy, π^2^/Ȓ^2^ could cause a blue shift with the decline of particle size, whereas A_1_/Ȓ and A_0_ would result in a red shift with the decreasing of particle size and rising of dielectric-constant ratio ϵ_1_/ϵ_2_ [21].

2 $$Eg\,=\,\frac{{\pi }^{2}}{{\hat R}^{2}}+ \frac{-8\pi {I}_{2}+4{I}_{3}-2{\pi }^{2}{\alpha }_{0}}{{\pi }^{2}}\frac{1}{\hat R}-{\bar \alpha}^{2}+ \varphi \left(\hat R\right)=\frac{{\pi }^{2}}{{\hat R}^{2}}+ \frac{{A}_{1}}{\hat R}+{A}_{0}+ \varphi \left(\hat R\right)$$

where Ȓ = R / a_B_^*^ (R is radius of nanoparticle, a_B_^*^ is the exciton Bohr radius in bulk material; The information of the other quantities in this equation could be found in Additional file 1: Eqs. S2.1–2.7 [21].

ZIF-8 showed the lowest λ_max_ (236 nm) and highest bandgap, meaning ZIF-8 needs more energy to generate electronic transition from full band to conducting band, which resulted in a UV absorption at low wavelength range and exerted a UV reflection at high wavelength range. The wide reflection of UVA, UVB and even some UVC could potentially endow ZIF-8 with a high anti-UV efficacy. So ZIF-8 was selected as the model MOFs for further study.

A widely used in vitro sun protection factor (SPF) assay with timesaving and no human subject involvement properties was performed to compare the photoprotective efficacy of ZIF-8, TiO_2_ and ZnO [22, 23]. ZIF-8 revealed an SPF value of 15.6, while which were 15.3 and 9.6 for TiO_2_ and ZnO, respectively, suggesting ZIF-8 could potentially provide a comparable or even higher UV protection effect relative to TiO_2_ and ZnO, respectively (Additional file 1: Fig. S16). Additionally, the SPF values of TiO_2_ and ZnO correlated well with that reported in the literatures, with 11.68 for TiO_2_ (15%) and 8.74 for ZnO (15%) [24, 25].

The digital photographs were taken under normal room light for the suspensions of TiO_2_ (15 wt%), ZnO (15 wt.%) and ZIF-8 (15 wt%) in glycerol. ZIF-8 yielded a much more transparent suspension in glycerol relative to that of TiO_2_ and ZnO, which is a competitive advantage from aesthetic considerations (Fig. 1B). ZIF-8 showed a low degradation in artificial sweat (36.2 ± 0.5% of degradation within 24 h) (Fig. 1E), which could potentially lower skin penetration and result in less time for ZIF-8 clearance. Nanoparticles would induce conduction band electron (e^−^) and valence band hole (h^+^) after UV radiation, which could further react with surrounding medium and produce free radicals of H^+^, H_2_O_2_, ·OH or HO_2_. Therefore, we studied the free radical formation of ZIF-8, TiO_2_, ZnO, MOF-5, IRMOF-1 and Zn_3_L_3_DMF_2_ using electron paramagnetic resonance (EPR) method. Interestingly, ZnO produced most free radical of ·OH (1.3 × 10^12^ spins/mm^3^), followed by TiO_2_ (5.9 × 10^11^ spins/mm^3^), MOF-5 (4.1 × 10^11^ spins/mm^3^), Zn_3_L_3_DMF_2_ (2.1 × 10^11^ spins/mm^3^), IRMOF-1 (6.2 × 10^10^ spins/mm^3^) and ZIF-8 (2.3 × 10^10^ spins/mm^3^) (Fig. 1F, Additional file 1: Fig. S15), suggesting ZIF-8 induced the least EPR signal compared to TiO_2_, ZnO, and the other Zn-based MOFs and exhibited weakest photocatalytic activity to generate hydroxyl radical, a hazard to human skin if topically applied. We also measured the temperature change and fluorescence production after the samples were exposed to UV, as the absorbed UV energy would be transferred into heat, fluorescence, or phosphorescence. Only 2–3 °C temperature increases were observed for all four groups compared to room temperature (RT) (Fig. 1H, I), suggesting that ZIF-8, TiO_2_ and ZnO would not do a second burn harm to the skin. Interestingly, a fluorescence at 621 nm was detected for ZIF-8 after UV exposure, indicating that the absorbed UV energy could partially be released as fluorescence for ZIF-8 (Fig. 1G). All these results above confirmed the UV reflection and absorption of MOFs could be tuned by changing the ligands. The selected MOFs, ZIF-8, showed appealing characteristics compared to commercial UV filters with wider and higher UV reflection, lower ROS production, and more transparent appearance.

### In vitro cytotoxicity

**Fig. 2:**
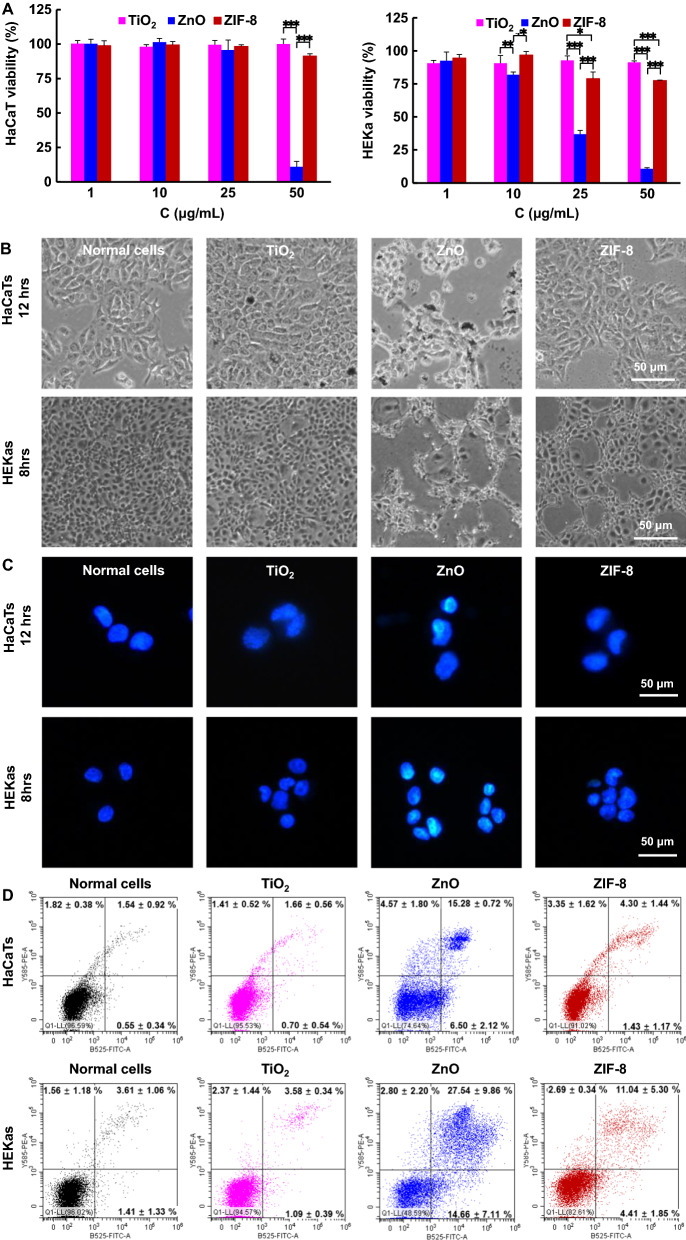
Toxicity on HaCaTs and HEKas. **A** Cytotoxicity of TiO_2_, ZnO and ZIF-8 at various concentrations toward HaCaTs and HEKas using MTT assay. **B** Digital images of HaCaTs and HEKas after treatment with TiO_2_, ZnO or ZIF-8. **C** Nucleus images with DAPI staining after cells were treated with TiO_2_, ZnO or ZIF-8. Obvious chromatin condensation was observed for ZnO-treated cells. **D** Flow cytometry analysis of cell apoptosis induced by TiO_2_, ZnO and ZIF-8

The toxicity of ZIF-8 to human immortalized epidermal keratinocytes (HaCaTs) and human epithelial keratinocytes (HEKas) cells was assessed using MTT (3-(4,5-dimethylthiazol-2-yl)-2,5-diphenyltetrazolium bromide) assay, a widely used tool to estimate the metabolic activity of living cells, where formazan with intense purple-blue color was formed from the lightly colored tetrazolium salt due to enzymatic reduction [26]. ZIF-8 showed much lower cytotoxicity compared to ZnO on both cell lines. The HaCaT survival rate was as high as 91.3 ± 1.6% for ZIF-8 at 50 µg mL^−1^, whereas which was only 11.0 ± 3.9% for ZnO at the same concentration. The survival rate of HEKas was decreased from 94.5 ± 2.8% to 79.0 ± 2.8% and 77.5 ± 0.3% for ZIF-8 at 10, 25, and 50 µg mL^−1^, respectively. On the contrary, the viability rate of cells treated with ZnO was 81.9 ± 2.2%, 36.8 ± 3.0%, and 10.8 ± 0.8%, respectively. No obvious cytotoxicity was observed for TiO_2_ at these concentrations (Fig. 2A). The cell apoptosis induced by these three nanoparticles also was evaluated by observing the cell and nucleus morphologies or by calculating cell apoptosis rates using flow cytometry. Obvious cell apoptosis with cell shrinkages and chromatin condensation was observed for both HaCaTs and HEKas after ZnO treatment at 60 μg mL^−1^ for 12 h or 8 h, respectively (Fig. 2B, C). Furthermore, the flow cytometry analysis showed that HaCaT apoptosis rates were 2.36%, 21.78%, and 5.73% after exposure with TiO_2_, ZnO and ZIF-8, respectively, whereas which were 4.66%, 42.20%, and 15.45% for HEKas (Fig. 2D), indicating that ZIF-8 caused much less cell apoptosis and could be more biocompatible relative to ZnO. However, it is still controversial for the cell toxicity of ZnO, because these assays do not take skin delivery into consideration [27]. Also, toxicity depends on exposure and toxic nature, ZnO toxicity could potentially not mean that much with absence of cellular exposure [10].

### Protection against DNA damage after UV exposure

**Fig. 3:**
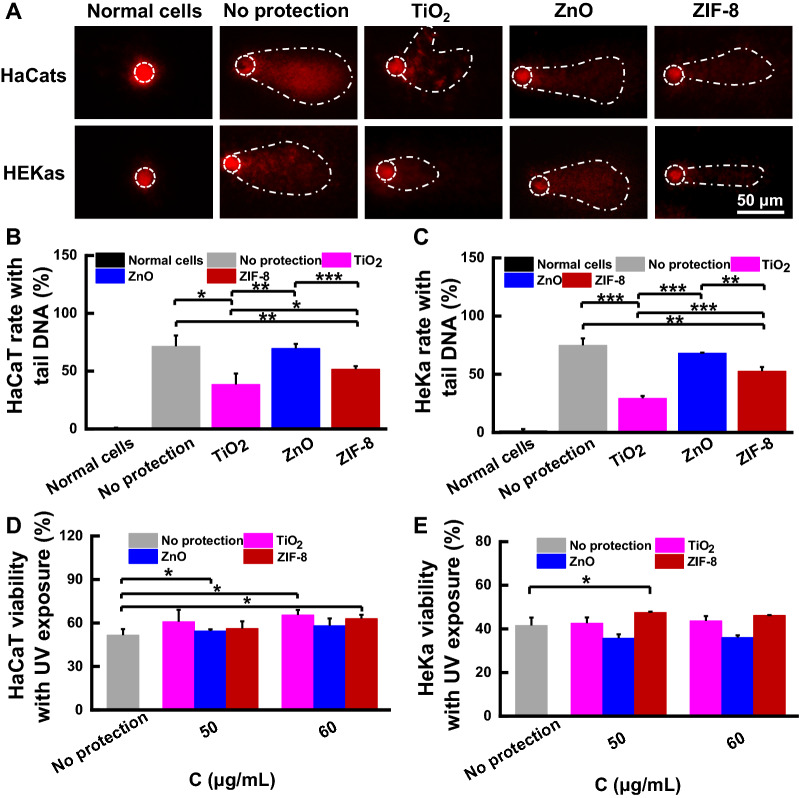
Photoprotective effects on HaCaTs and HEKas. **A** Representative fluorescence images of cells with DNA tail after pre-protection and subsequent UV exposure. **B**, **C** Quantitative analysis of (**B**) HaCaTs and (**C**) HEKas with DNA tail. **D**, **E**) Cell viability of (**D**) HaCaTs and **E** HEKas with pre-protection and subsequent UV exposure

UVB is reported to induce oxidative DNA damage through the generation of free radicals and ROS [2, 28]. In order to test the protective effect of ZIF-8 against UVB, comet assay was performed, and DNA damage was quantified. Without protection, 71.5 ± 9.3% of HaCaTs possessed DNA tails (single-strand or double-strand DNA breaks), whereas which was significantly reduced to 51.7 ± 3.5% with ZIF-8 protection and 38.5 ± 9.5% with TiO_2_ protection. HEKas showed a similar result, with 74.9 ± 6.0% for no protection group but 52.7 ± 3.6% and 29.4 ± 2.1% for ZIF-8 and TiO_2_ groups, respectively (Fig. 3A–C). These results confirmed ZIF-8 could efficiently prevent DNA breaks in skin cells after UV exposure, possibly due to its high UV reflection capability. TiO_2_ also exerted high DNA protection effects, potentially caused by its high UV absorption [29]. ZnO did not show protective effect against DNA fragmentation, which may be due to UV exposure accelerated the entrance of ZnO to skin cells, resulted in more ROS production due to the increased ZnO level and finally DNA damage [28].

The UV shielding capability of ZIF-8, TiO_2_, and ZnO also was assessed using MTT assay. TiO_2_ significantly increased HaCaT viabilities to 60.9 ± 8.2% at 50 μg mL^−1^ and 65.6 ± 3.4% at 60 μg mL^−1^, compared to cells without protection (51.6 ± 4.2%). ZIF-8 also exerted a protective effect for HaCats, with 63.0 ± 2.7% cell viability at 60 μg mL^−1^. Unfortunately, ZnO did not protect HaCaTs against UV irradiation, as shown by negligible changes on cell viabilities. For HEKas, only ZIF-8 at 50 μg mL^−1^ revealed an obviously higher cell viability with 47.6 ± 0.3%, compared to non-protection group (41.6 ± 3.6%) (Fig. 3D, E). ZIF-8, TiO_2_ and ZnO were not toxic in this test, because the nanoparticles were washed away using PBS and the cells were cultured in fresh medium without nanoparticles after UV exposure. These results confirmed that both TiO_2_ and ZIF-8, but not ZnO, could efficiently protect cells against UV exposure.

### Protection against ROS generations in skin cells

ROS levels in HaCaTs and HEKas were measured using confocal fluorescent microscopy and flow cytometry after UVB or UVA exposures. After UVB irradiation, HaCaTs and HEKas with ZIF-8 pretreatment showed no obvious or less increase of ROS relative to that of the cells without protection or with the protections of TiO_2_, ZnO, MOF-5, IRMOF-1 or Zn_3_L_3_DMF_2_ (Additional file 1: Figs. S19, 20). After UVA exposure, both cells revealed similar results, with few or less ROS generations in ZIF-8 group, whereas which were elevated in the groups of No protection, TiO_2_, ZnO, MOF-5, IRMOF-1 and Zn_3_L_3_DMF_2_ (Additional file 1: Figs. S21, 22). All these results confirmed ZIF-8 could effectively reduce ROS productions in skin cells with UVA or UVB exposures.

### Protective effects on mouse skin

**Fig. 4:**
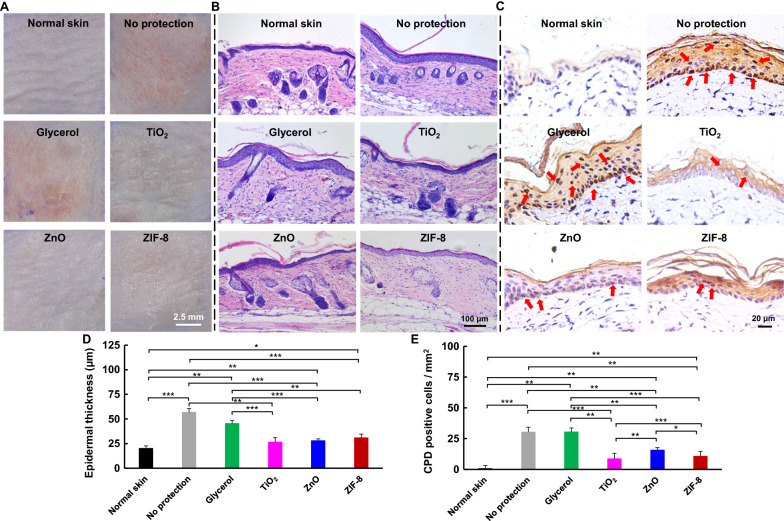
Photoprotective effects on mouse skin. **A** Representative digital graphs of mouse dorsal skin three days after pre-protection and subsequent UV exposure. ZIF-8 group showed less erythema compared to no protection or glycerol groups. **B** Microscope photographs of skin with H&E staining. **C** Microscope photographs of skin with CPD immunohistochemistry. **D**, **E** Quantitative analysis of (D) epidermal thickness and **E** CPD positive cells in the skin after pre-protection and subsequent UV exposure. ZIF-8 inhibited epidermal hyperplasia and CPD formation

The anti-UV effects of ZIF-8 was assessed on the dorsal skin of BALB/c mice, which was divided into five parts (1 cm × 1 cm for each part), preincubated with either glycerol, ZnO, TiO_2_, or ZIF-8 at an optimized dose of 15% for 15 min, and then exposed to UVB (280—320 nm) at an optimized UV dose of 206 mJ m^−2^. The skin with no protection was used as control (Additional file 1: Figs. S23, 24). After three days, the skin with treatments of ZIF-8, ZnO and TiO_2_ showed much less ulceration, edema or erythema compared to that of glycerol or no protection groups (Fig. 4A, Additional file 1: Fig. S25), suggesting ZIF-8, ZnO and TiO_2_ are all protective against macroscopic skin damages from UVB. Also, the skin histological assay by H&E staining revealed that the epidermal thickness was significantly increased after UV exposure from 20.1 ± 2.6 μm for normal skin to 56.6 ± 4.1 μm and 45.5 ± 3.2 μm for no protection and glycerol groups, respectively. However, the epidermal thickness increase was significantly inhibited by TiO_2_, ZnO and ZIF-8 treatments, with an epidermal thickness of 26.5 ± 4.6 μm, 27.9 ± 2.1 μm, and 30.8 ± 4.1 μm, respectively. No obvious difference for epidermal thickness was observed for these three groups (Fig. 4B, D), confirming ZIF-8 achieved comparable anti-epidermal hyperplasia effects to TiO_2_ and ZnO on mouse skin, which would contribute to a smoother skin appearance.

UVB irradiation induces the formation of cyclobutane pyrimidine dimers (CPDs), causes DNA lesions, blocks normal DNA replication if DNA damage is not repaired and leads to mutagenesis and even carcinogenesis [30]. So CPDs formed in the skin after UV irradiation was stained using immunohistochemistry (IHC). Obvious CPDs were observed for the skin without protection (30.3 ± 0.5 CPD^+^ cells per mm^2^) and glycerol pretreatment (30.4 ± 4.1 CPD^+^ cells per mm^2^), but which was obviously reduced for the skins with ZIF-8, ZnO or TiO_2_ protections (10.6 ± 2.5, 15.7 ± 2.1, or 8.6 ± 0.5 CPD^+^ cells per mm^2^, respectively). ZIF-8 even achieved a significant reduction of CPD compared to ZnO, confirming ZIF-8 could protect skin against UV-caused DNA damage on mouse skin (Fig. 4C, E). The lowered CPD formation could be due to the reduced ROS formation for the skin with ZIF-8 shielding. After UVB irradiation, the ROS level was (2.4 ± 0.3) × 10^5^ for the skin with ZIF-8 protection, which was significantly lower relative to that of TiO_2_ ((3.4 ± 0.6) × 10^5^) and ZnO ((4.4 ± 1.1) × 10^5^) groups, respectively, and even comparable to that of normal skin ((2.5 ± 0.2) × 10^5^) without UVB exposure, suggesting ZIF-8 could effectively protect the skin against ROS generation caused by UVB irradiation (Additional file 1: Fig. S26).

Collagen is an essential component to maintain skin structure, and the degradation of which after UV radiation would cause wrinkling and laxity [31]. Thus, we performed Masson’s trichrome staining for paraffin sections of the skin. Compared to normal skin, a reduced collagen density and irregularly distributed collagen fiber were observed after UV exposure for no protection and glycerol groups, but not for ZIF-8, TiO_2_ and ZnO groups, confirming ZIF-8 exerted a similar protective effect to TiO_2_ and ZnO against collagen degradation induced by UV irradiation (Additional file 1: Fig. S27A). The collagen protective effect of ZIF-8 would be beneficial to the maintenance of a smooth skin after UV exposure.

UVB exposure is also reported to cause pro-inflammatory responses with elevated levels of pro-inflammatory cytokines (such as IL-1β, TNF-α and IL-6) and lead to inflammatory disequilibrium in the skin [32–35]. We found that IL-1β expression in the skin was increased for no protection and glycerol groups after UV exposure, while which was not observed for ZIF-8, TiO_2_ and ZnO groups, suggesting ZIF-8 could inhibit IL-1β expression, thereby mitigating pro-inflammatory responses (Additional file 1: Fig. S27B). Overexpression of IL-1β would result in overproduction of MMP-2, MMP-9 and MMP-12, which would further lead to the degradation of elastin and collagens I, II and III [32, 36–38]. The inhibition of ZIF-8 on IL-1β overexpression would contribute to the maintenance of collagen morphology and well collagen distribution in the skin, endowing a smooth, full and firm skin appearance.

### Protective effects on pig skin

**Fig. 5:**
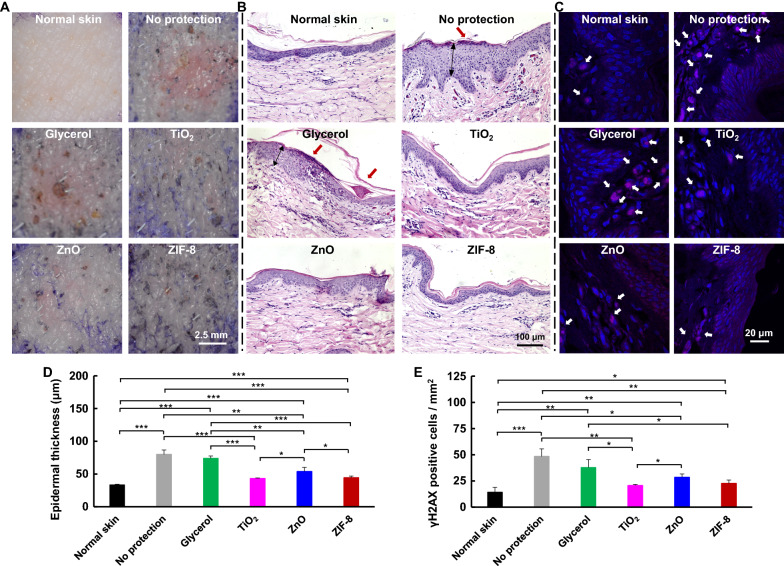
Photoprotective effects on pig skin. **A** Representative digital images of pig skin after pre-protection and subsequent UV exposure. Fewer erythema was observed for ZIF-8 group, compared to no protection group. **B** Photographs of skin with H&E staining after pre-protection and subsequent UV irradiation. (Red arrows point to parakeratosis.) **C** γ-H_2_AX immunofluorescence images of skin sections after pre-protection and subsequent UV exposure. (Nucleus: blue; γ-H_2_AX: red; White arrows point to γ-H_2_AX^+^ cells.) **D**, **E** Quantitative analysis of (**D**) epidermal thickness and (**E**) γ-H_2_AX positive cells on pig skin with pre-protection and subsequent UV exposure. ZIF-8 inhibited epidermal hyperplasia and γ-H_2_AX formation

In order to evaluate the clinical translational potential of ZIF-8 as sun-screening agent, we further investigated the protective effect of ZIF-8 on Ba-Ma miniature pig, which shows similar skin histology compared to that of human in terms of horny layer thickness, epidermal and dermal thicknesses, epidermis/dermis ratio, and collagen physicochemical properties [39]. Also, Ba-Ma miniature pig exhibits some special characteristics, including heterogeneity of basal cells, granules of mast cells, serrated pattern for epidermal-dermal interface, and developed vascular system, which are found only on the skin of humans, nonhuman primates, and pigs [39]. The dorsal skin of Ba-Ma miniature pig was demarcated into squares (1 cm × 1 cm), randomly pretreated with glycerol, TiO_2_, ZnO, or ZIF-8 for 15 min, and exposed to UVB radiation at the optimized dose (544 mJ cm^−2^) with an optimized ZIF-8 dose of 15% (Additional file 1: Figs. S28, 29). After UV exposure, the skin with protections of ZIF-8, TiO_2_, or ZnO exhibited much less erythema compared to that of the skin without protection or with protection of glycerol (Fig. 5A, Additional file 1: Fig. S30). Also, the epidermal layer thickness was increased from 33.5 ± 0.9 μm for normal skin to 79.9 ± 6.7 μm or 74.1 ± 3.6 μm for no protection or glycerol groups. However, the epidermal layer thickness was significantly lowered by TiO_2_ (43.1 ± 0.9 μm), ZnO (53.7 ± 6.6 μm), or ZIF-8 (44.4 ± 2.5 μm) groups, suggesting all of them could inhibit the epidermal hyperplasia after UV exposure. Interestingly, the epidermal thickness of ZIF-8 group was similar to that of TiO_2_, which was much thinner than that of ZnO group, confirming ZIF-8 achieved comparable anti-epidermal hyperplasia effects to TiO_2_ and even better effects than ZnO (Fig. 5B, D).

UV exposure would induce skin DNA damage with the formation of DNA double-strand breaks (DSB), which further induces the phosphorylation of histone H_2_AX on serine 139 [40]. Thus, γ-H_2_AX formation is considered as a DSB-response marker and was assessed using immunofluorescence staining. UV irradiation induced remarkable γ-H_2_AX on no protection skin and glycerol pretreated skin, with 48.4 ± 7.3 and 37.8 ± 7.7 γ-H_2_AX positive (γ-H_2_AX^+^) cells per mm^2^, respectively. However, after pretreatment with TiO_2_, ZnO or ZIF-8, γ-H_2_AX^+^ cells were significantly reduced to 20.6 ± 1.2, 28.5 ± 3.2, and 22.6 ± 3.2 per mm^2^ (Fig. 5C, E), suggesting ZIF-8 achieved comparable effects to TiO_2_ and ZnO on inhibiting UV-induced DNA damage.

Porcine skin is confirmed to be a suitable model for sunscreen protection efficacy assessment because the universal sun protection factor obtained using porcine skin correlates well with that on human skin (correlation factor R^2^ = 0.98) [41]. The comparable or even higher anti-UV efficacy of ZIF-8 relative to TiO_2_ or ZnO on pig skin suggests that ZIF-8 could be a potential effective sunscreen surrogate for human.

### Long-term in vivo toxicity

**Fig. 6:**
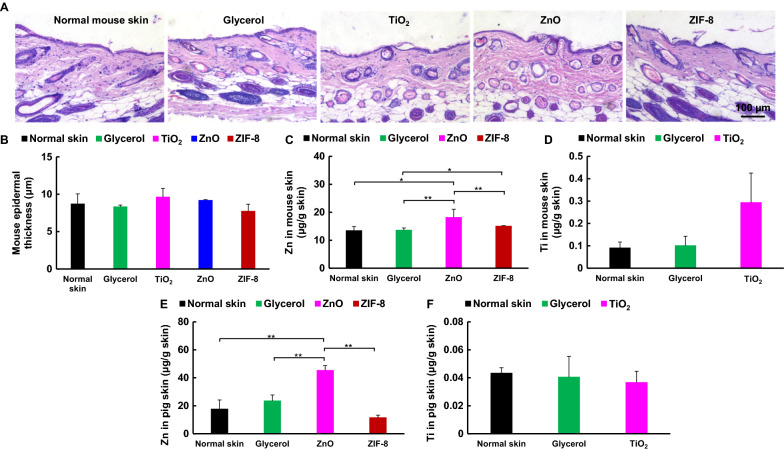
Long term toxicity and penetration into mouse or pig skins. After repeated dosing for total six times in half a month, the skin was collected and subjected for H&E staining. **A** Representative H&E staining images of mouse skin. **B** Quantitative analysis of epidermal thickness. No obvious epidermal thickness was increased after long-term application of ZIF-8. **C**–**F** Penetrations of (**C**, **E**) ZIF-8, ZnO, and (**D**, **F**) TiO_2_ into (**C**, **D**) mouse and **E**, **F** pig skin. Skin was treated with glycerol, ZIF-8, ZnO, and TiO_2_ for 6 h, then the Zn^2+^ and Ti^4+^ in skin were detected by ICP-MS. ZnO easily penetrated mouse and pig skin, but not for ZIF-8 and TiO_2_

We studied the long-term toxicity by applying either glycerol, TiO_2_, ZnO or ZIF-8 to mouse dorsal skin once every other day for a total of 6 applications. The skin showed no obvious signs of acute histology toxicity or long-term inflammation for all four groups. The epidermis structures, including corneum structure, epidermis thickness, skin follicles and sebaceous structure, also were not affected for all groups (Fig. 6A, B). Also, the liver function and kidney function were normal and the tissues, including heart, liver, spleen, lung, and kidney, were not affected after treatments with TiO_2_, ZnO, or ZIF-8 (Additional file 1: Figs. S31, 32).

We further investigated the penetration of TiO_2_, ZnO, or ZIF-8 into mouse or pig skin and their accumulations in mouse blood, heart, liver, spleen, lung, and kidney by measuring Zn or Ti contents remaining in the tissues using inductively coupled plasma-mass spectrometry (ICP-MS). The normal Zn content in mouse skin was 13.5 ± 1.5 μg g^−1^ tissue, which was significantly increased to 18.2 ± 3.0 μg g^−1^ tissue for ZnO group. This correlates well with previous reports that Zn ions dissociated from ZnO could get access into human skin after multiple uses [10, 42]. However, the Zn content in mouse skin was kept at normal level after ZIF-8 treatment (15.0 ± 0.2 μg g^−1^) (Fig. 6C), possibly due to its hydrophobic surface [43]. No obvious increases of Zn level were observed in mouse blood and main organs (Additional file 1: Fig. S33). The Ti content in normal mouse skin with no treatments was 0.10 ± 0.03 μg g^−1^ tissue, however, which was slightly but not significantly increased to 0.30 ± 0.14 μg g^−1^ tissue after TiO_2_ exposure (Fig. 6D). Ti levels in blood and main organs were not affected with TiO_2_ treatment. (Additional file 1: Fig. S33). We also confirmed skin penetrations on pig, a model for human skin due to the similar skin morphology and permeability characteristics [44], which yielded similar results under the same treatment. The Zn level for ZnO group was significantly increased to 45.2 ± 3.5 μg g^−1^ tissue from 17.6 ± 6.6 μg g^−1^ tissue in normal pig skin. There was no obvious Zn level increase in the pig skin with glycerol (23.5 ± 4.3 μg g^−1^ tissue) or ZIF-8 (11.5 ± 1.8 μg g^−1^ tissue) treatments (Fig. 6E). TiO_2_ also did not increase Ti level on pig skin, compared to no treatment or glycerol groups (Fig. 6F). The lower skin penetration of ZIF-8 was potentially caused by its hydrophobicity [43], which would potentially contribute to a lower topical toxicity due to the low ZIF-8 level in skin, and further result in a lower systemic risk with less Zn ions in circulation before ZIF-8 was cleared completely.

## Conclusions

Exposure to solar UV is still a significant health risk for human, and there is still urgent need for safe and effective sunscreens due to the safety and efficacy concerns of current commercially available sunscreens. Herein, we synthesize a serial of Zinc-based MOFs and confirm the UV absorption and reflection can be tuned by choosing different organic ligands. Due to the wide and high UV reflection, ZIF-8 is selected as model MOFs and the potential as UV filter is evaluated. ZIF-8 exhibits good biocompatibility, low radical production, weak skin penetration, and achieves a high anti-UV efficacy on both mouse and pig skin, respectively, suggesting ZIF-8 could be a potential sunscreen surrogate for human with high efficacy and safety. All these results demonstrate that Zinc-based MOFs could potentially be a suitable platform to develop sunscreens through tuning UV reflectance and other characteristics, such as hydrophobicity, stability, and photocatalytic activity.

## Experimental section

### Materials and animals

Zinc oxide (ZnO, ≥ 99.9%), titanium oxide (TiO_2_, ≥ 99.9%), zinc acetate dihydrate (Zn(Ac)_2_·2H_2_O, ≥ 99.9%), terephthalic acid (H_2_BDC, 99%), N,N-dimethylformamide (DMF, 99.8%) and 2-methylimidazole (2-MeIM) were obtained from J&K Chemicals (Beijing, China). Dimethyl sulfoxide-d_6_ (DMSO-d_6_, ≥ 99.9%) were purchased from Tian in Fuyu Fine Chemical Co., Ltd (Tianjin, China). 4,4’-stilbenedicarboxylic acid (LH_2_) was bought from Flurochem (UK). Dulbecco’s modified eagle medium (DMEM), minimum essential medium (MEM) and fetal bovine serum (FBS) were purchased from Thermo Fisher Scientific (Waltham, MA, USA). Thiazolyl blue (MTT) was bought from Biosharp (Hefei, China). IL-1β polyclonal antibody was obtained from Signalway Antibody LLC (College Park, USA). Phospho-histone H_2_AX (Ser139) antibody (γ-H_2_AX) was bought from Affinity Biosciences (Cincinnati, USA). Anti-thymine dimer antibody (cyclobutane pyrimidine dimers, CPDs) was obtained from Sigma-Aldrich (Louis, USA). Masson’s trichrome staining kit was purchased from Beijing Solarbio Science & Technology (Beijing, China). Annexin V-FITC/PI apoptosis detection kit was bought from Procell Life Science & Technology (Wuhan, China). 2,7-Dichlorofluorescein Diacetate (DCFH-DA) probe kit and DNA damage assay kit were obtained from Nanjing Jiancheng Bioengineering Institute (Nanjing, China). UVB light with a peak emission at 308 nm was bought from Sankyo Denki Co. (Tokyo, Japan).

Human immortalized epidermal keratinocytes (HaCaTs) were purchased from China Center for Type Culture Collection (CCTCC, Wuhan, China) and cultured in DMEM with 10% FBS and 1% antibiotics. Human epithelial keratinocytes (HEKas) were obtained from Jennio Biotech (Guangzhou, China) and reserved in MEM medium with 10% FBS and 1% antibiotics. Both cells were placed in a humidified incubator with 5% CO_2_ at 37 °C.

Male BALB/c mice (6 weeks) were obtained from Laboratory Animal Centre of Guangzhou University of Chinese Medicine. Male Ba-Ma miniature pig (2–3 months) was bought from Dongguan Songshan Lake Laboratory Animal Technology Co., Ltd (Guangdong, China). All the protocols for animal experiments were approved by the Animal Ethics Committee of Southern Medical University, China. (Approval number of the laboratory: L2018244).

### Instruments and methodologies

Powder X-ray diffraction (PXRD) was performed on Bruker D8 advance (Bruker Corporation, Billerica, USA) at the speed of 10° min^−1^ with an angle range of 5°–60°. ^1^H NMR spectra were recorded on 400 MHz Bruker (Bruker Corporation, Billerica, USA). Fourier transform infrared (FT-IR) spectra from KBr pellets were carried out using a Nicolet iS10 spectrometer (Thermo, Waltham, MA, USA). Elemental analysis was performed on an Elementar Vario-EL Cube CHNS elemental analyzer (Vario EL cube, Hanau, Germany). Transmission electron microscope (TEM) imaging was acquired on Hitachi H-7650 microscope (80 kV, Hitachi, Tokyo, Japan). N_2_ isotherm measurements were carried out on ASAP 2460 (Micromeritics instrument Ltd., GA, Norcross, USA) at 77 K with 20–100 mg of sample per measurement. Inductively coupled plasma mass spectrometry (ICP-MS) was performed on Agilent 7700 (Agilent Technologies, Inc., Santa Clara, USA). X-ray photoelectron spectroscopy (XPS) were measured using K-Alpha (Thermo Scientific, Waltham, MA, USA). Thermogravimetric analysis (TGA) was acquired on TGA 5500 from 30 to 780 °C at a speed of 5 °C min^−1^ (TA Instruments, New Castle, USA). UV–visible diffuse-reflectance spectrum were performed from BaSO_4_ pellets on Lambda 950 (PerkinElmer Inc., Waltham, USA) equipped with photometric integrating sphere (150 mm Int. sphere). The zeta potentials of Zn-based MOFs were measured using Zetasizer Nano ZS (Malvern Panalytical, UK). To measure electron paramagnetic resonance (EPR), TiO_2_, ZnO, or ZIF-8 (2 mL, 800 μg mL^−1^) were mixed with free radical catcher α-(4-Pyridyl N-oxide)-N-tert-butylnitrone (POBN, 38.8 mg), irradiated with UV (200–400 nm) for 10 min at 25 ± 0.1 °C under standard atmospheric pressure, and analyzed using a A300 spectrometer (Bruker Corporation, Billerica, USA). The samples at 800 μg mL^−1^ but not 50 μg mL^−1^, were selected for EPR measurement, because ROS signal was too low to be detected at 50 μg mL^−1^, though ZIF-8 and TiO_2_ could protect skin cells against UV damage at 50 μg mL^−1^. (Additional file 1: Fig. S14).

### The synthesis and characterization of MOFs

*ZIF-8:* ZIF-8 with various sizes were synthesized according to previously reported methods with minor modifications [45, 46]. Briefly, Zn(Ac)_2_·2H_2_O (2.28 × 10^–1^ mol L^−1^ in DMF, 2 mL) were added with various ratios of Zn^2+^ to 2-MeIM (4 mL of DMF, 2.28 × 10^–1^ mol L^−1^ for ZIF-8 1:2, 5.71 × 10^–1^ mol L^−1^ for ZIF-8 1:5, 9.14 × 10^–1^ mol L^−1^ for ZIF-8 1:8, 18.28 × 10^–1^ mol L^−1^ for ZIF-8 1:16) under stirring at 450 rpm to synthesize ZIF-8 with different sizes. After 7.5 h, the reaction solution was centrifuged at 8000 rpm for 2 min and washed with DMF (5 mL) and ethanol (5 mL) for 2 times, respectively. The samples were resuspended with ethanol and stored at – 80 °C for further use.

TEM, ^1^H NMR, PXRD, XPS, and N_2_ isotherm were conducted to confirm the structure of ZIF-8.

*ZIF-8 1:2:* ^1^H NMR (DMSO-d_6_/D_2_SO_4_ (9:1, v/v)): 7.49 (s, 2H, Imidazole H), 2.60 (s, 3H, –CH_3_). Yield: 29.7%. BET surface area was 1237.8 m^2^ g^−1^. Pore size: 9.3–15.0 Å. XPS data also revealed the successful synthesis of ZIF-8 1:2 (Additional file 1: Fig. S3).

*ZIF-8 1:5:* ^1^H NMR (DMSO-d_6_/D_2_SO_4_ (9:1, v/v)): 7.49 (s, 2H, Imidazole H), 2.56 (s, 3H, –CH_3_). Yield: 19.1%. BET surface area was 1306.9 m^2^ g^−1^. Pore size: 9.3–15.0 Å. XPS data also revealed the successful synthesis of ZIF-8 1:5 (Additional file 1: Fig. S4).

*ZIF-8 1:8:* ^1^H NMR (DMSO-d_6_/D_2_SO_4_ (9:1, v/v)): 7.38 (s, 2H, Imidazole H), 2.48 (s, 3H, –CH_3_). Yield: 19.0%. BET surface area was 1566.8 m^2^ g^−1^, which was similar to the reported values, potentially due to the high crystallinity and the excellent activation before N_2_ isotherm measurement. [47, 48] Pore size: 9.3–16.0 Å. XPS data also revealed the successful synthesis of ZIF-8 1:8 (Additional file 1: Fig. S5).

*ZIF-8 1:16:* ^1^H NMR (DMSO-d_6_/D_2_SO_4_ (9:1, v/v)): 7.49 (s, 2H, Imidazole H), 2.50 (s, 3H, –CH_3_). Yield: 15.7%. BET surface area was 1267.4 m^2^ g^−1^. Pore size: 9.3–15.9 Å. XPS data also revealed the successful synthesis of ZIF-8 1:16 (Additional file 1: Fig. S6).

ZIF-8 was observed using TEM and the particle sizes were plotted thereafter. The particle size were 164.8 ± 32.6 nm, 102.5 ± 26.8 nm, 82.3 ± 24.5 nm, and 80.0 ± 37.7 nm for ZIF-8 1:2, ZIF-8 1:5, ZIF-8 1:8, ZIF-8 1:16, respectively, confirming the size was decreased with the ratios of Zn^2+^ to 2-MeIM decreasing (Fig. 1A, Additional file 1: Figs. S1, S7A, B). These were similar to the previous report that ZIF-8 sizes were decreased from 110 to 40 nm with the ratios of Zn^2+^ to 2-MeIM decreasing from 1:2 to 1:16 [46]. However, no obvious ZIF-8 morphology variations were observed, (Fig. 1A, S7A) which were not consistent with the reports that morphologies of ZIF-8 turned from cube to sphere with the ratios of Zn^2+^ to 2-MeIM decreasing [46]. Possibly due to the decline of particle sizes, UV reflectance, especially for UVB and UVC, was enhanced with the decrease of Zn^2+^ to 2-MeIM ratios, which reached to the highest value for ZIF-8 1:8. No further enhancement was observed for ZIF-8 1:16, (Additional file 1: Fig. S7C) possibly because size of ZIF-8 1:16 was similar to that of ZIF-8 1:8. So ZIF-8 1:8 was selected as model ZIF-8 in the following experiments.

To study the energy release ways of ZIF-8 after UV exposure, TiO_2_, ZnO, or ZIF-8 (150 mg mL^−1^ in glycerol, 1 mL) were irradiated with UVB at the dose of 6.408 × 10^4^ J m^−2^, thermal images were then taken by FLIR C2 Compact Thermal Camera (FLIR Systems, Wilsonville, USA). After irradiation, the fluorescence intensity of TiO_2_, ZnO, and ZIF-8 (50 μg mL^−1^ in ethanol) were obtained by F97 pro fluorescence spectrophotometer (Lengguang Tech., Shanghai, China) with excitation wavelength at 308 nm.

TEM, PXRD, XPS also were used to confirm the structures of TiO_2_ and ZnO (Fig. 1A, Additional file 1: Figs. S8, S9).

*MOF-5:* MOF-5 was synthesized as previously reported [49]. Zn(NO_3_)_2_·6H_2_O (290 mg, 1 mmol) was added into H_2_BDC (5.0 × 10^–2^ mol L^−1^ in DMF, 10 mL) and heated at 120 °C for 21 h. The reaction solution was centrifuged at 8000 rpm for 2 min and washed with DMF (5 mL) for 3 times. The sample was stored in ethanol at − 80 °C for further use. ^1^H NMR (DMSO-d_6_/D_2_SO_4_ (9:1, v/v)): 8.07 (s, 4H, Ar H). Yield: 14.2%. BET surface area: 741.5 m^2^ g^−1^. Pore size: 6.5—15.0 Å. XPS data confirmed the successful synthesis of MOF-5 (Additional file 1: Figs. S10, S13).

*IRMOF-1:* IRMOF-1 was synthesized according to previously reported method [50, 51]. Briefly, Zn(NO_3_)_2_·6H_2_O (10 mg, 0.034 mmol) was added into H_2_BDC solution (2.7 × 10^–2^ mol L^−1^ in DMF, 10 mL), and heated at 100 °C for 18 h. The crystals were centrifuged at 8000 rpm for 2 min and washed with DMF (5 mL) and ethanol (5 mL) for 3 times, respectively. The sample was stored in ethanol at − 80 °C for further use. ^1^H NMR (DMSO-d_6_/D_2_SO_4_ (9:1, v/v)): 8.07 (s, 4H, Ar H). Yield: 55.2%. BET surface area: 799.2 m^2^ g^−1^. Pore size: 6.0–25.0 Å. XPS data also revealed the successful synthesis of IRMOF-1 (Additional file 1: Figs. S11, S13).

*Zn*_*3*_*L*_*3*_*(DMF)*_*2*_*:* Zn_3_L_3_(DMF)_2_ was synthesized following previously reported methods [12]. Zn(NO_3_)_2_·6H_2_O (209.2 mg, 0.7 mmol) was added to LH_2_ (9.14 × 10^–1^ mol L^−1^ in DMF, 20 mL), heated at 75 °C for 16 h and followed by heating at 85 °C for 4 h. Thereafter, the samples were centrifuged at 8000 rpm for 2 min and the precipitate was washed with DMF (5 mL) and ethanol (5 mL) for 3 times, respectively. The sample was stored in ethanol at − 80 °C for further use. ^1^H NMR (DMSO-d_6_/D_2_SO_4_ (9:1, v/v)): 7.94 (s, 2H, Ar H), 7.82 (s, 2H, Ar H), 7.49 (s, 2H, C=CH), 2.93 (S, –CH_3_, 3H), 2.77 (S, 3H, –CH_3_). Yield: 12.8%. BET surface area: 910.1 m^2^ g^−1^. Pore size: 6.4–11.8 Å. XPS data also revealed the successful synthesis of Zn_3_L_3_(DMF)_2_ (Additional file 1: Fig. S12, 13).

*The degradations of MOFs:* The degradation of Zn-based MOFs in artificial sweat was assessed as previously reported. Briefly, ZIF-8 1:8 (10 mg, 0.5 mg), MOF-5 (0.5 mg), IRMOF-1 (0.5 mg), and Zn_3_L_3_(DMF)_2_ (0.5 mg) dispersed in glycerol were sealed into dialysis tubes, followed by an incubation in artificial sweat (0.5% NaCl, 0.1% lactic acid, and 0.1% urea, pH = 6.5, 32 °C ) at 100 rpm. At the predetermined time intervals, artificial sweat samples were collected and renewed with fresh media. The MOF ligands in artificial samples were measured with NanoPhotometer (NP80 Touch, IMPLEN, Germany). ZIF-8 (10 mg) showed the lowest degradation rate (36.2 ± 0.5% of degradation within 24 h), followed by ZIF-8 (0.5 mg) (46.6 ± 6.2% in 4 h, 72.0 ± 8.3% in 24 h), Zn_3_L_3_DMF_2_ (0.5 mg) (46.8 ± 6.8% in 4 h, 80.7 ± 9.2% in 24 h), MOF-5 (0.5 mg) (78.3 ± 5.0% in 4 h), and IRMOF-1 (0.5 mg) (86.8 ± 5.9% in 4 h) (Fig. 1E, Additional file 1: Fig. S13H).

*In vitro SPF measurement:* TiO_2_, ZnO, and ZIF-8 were dispersed in ethanol (15% by weight), respectively, and treated with ultrasonication for 10 min. The absorption at 290–320 nm was assessed using NanoDrop 1000 UV–VIS spectrophotometer (Additional file 1: Fig. S16). SPF values were calculated according to Mansur Eq. (3) below [22]:

3 $${SPF}_{\mathrm{spectrophotometric}}=\mathrm{CF} \times \sum_{290}^{320}\mathrm{EE }\left(\uplambda \right)\times \mathrm{I }\left(\uplambda \right)\times \mathrm{Abs }\left(\uplambda \right)$$

where CF = correction factor (10), EE (λ) = erythemal effect caused by the radiation with λ wavelength, I (λ) = solar intensity with λ wavelength, Abs (λ) = absorbance of samples. EE × I are constants as previously reported [23].

*In vitro cytotoxicity assay* HaCaTs or HEKas were seeded (2 × 10^4^ cells per well) into 96-well plates and incubated for 24 h. Thereafter, the cells were treated with saline or various concentrations (1—100 μg mL^−1^) of TiO_2_, ZnO, or ZIF-8 for 24 h and then incubated with MTT solution (0.5 mg mL^−1^) at 37 °C for 4 h. Finally, DMSO (200 μL) was added to dissolve the resulted crystal and the absorbance at 570 nm were measured using a microplate reader (Multiskan FC, Thermo Scientific, Waltham, MA USA). Cell viability was expressed as a percentage of the absorbance to that of the control experiment without treatment.

*Cell apoptosis assay* Cells were seeded into 12-well plates (3 × 10^5^ cells per well) and incubated for 24 h. After treated with TiO_2_, ZnO, or ZIF-8 (60 μg mL^−1^) (HaCaTs for 12 h and HEKas for 8 h), cells were photographed using a microscope with white light. Thereafter, cells were harvested, fixed with 4% paraformaldehyde at 4 °C for 30 min, permeabilized in 1% Triton-X 100 for another 30 min, and then stained with DAPI for 20 min. The stained cells were examined using fluorescence microscope (DMI8, Leica, Germany).

Cell apoptosis also was assessed using flow cytometry. Cells were grown in 6-well plates at a density of 1 × 10^6^ cells per well and incubated to complete adhesion. Then, the cells were treated with TiO_2_, ZnO or ZIF-8 (60 μg mL^−1^, HaCaTs for 12 h and HEKas for 8 h). Thereafter, the cells were detached with trypsin, centrifuged at 300 g for 5 min, stained with Annexin V-FITC and propidium iodide (PI) for 20 min, and analyzed by flow cytometer (CytoFLEX LX, Beckman Coulter Biotechnology Co., California, USA).

*Protection against UV-induced cell death* The cell viability of HaCaTs or HEKas after exposure with various UV doses for 24 h was first assessed by MTT assay. Around 50% cell growth inhibition was achieved at UV doses of 35 mJ cm^−2^ for HaCaTs and 75.6 mJ cm^−2^ for HEKas (Additional file 1: Fig. S17). Also, the optimized UV doses are similar to the previously reported UVB irradiation doses (30 or 50 mJ cm^−2^) for cells [52, 53]. Thus, the two UV doses were used in the following protection experiments. HaCaTs or HEKas (30 μL of medium per well) in 96-well plates were treated with TiO_2_, ZnO or ZIF-8 at concentrations of 50 μg mL^−1^ or 60 μg mL^−1^ for 15 min, irradiated with UV lamp (emission peak 308 nm, Sankyo Denki Co., Taiwan, China) at the optimized doses, washed with PBS to remove the nanoparticles and incubated in fresh complete medium for another 24 h. The cell viability was then assessed using MTT assay.

*Protection against DNA damage caused by UV irradiation* Comet assay was used to determine the photoprotective effect of ZIF-8 to HaCaTs or HEKas. To optimize UV doses, cells were seeded into 12-well plates (5 × 10^5^ cells per well) and incubated for 24 h. Thereafter, cells were exposed to four UV doses (84, 98, 114 or 126 mJ cm^−2^ for HaCaTs and 38.5, 52.5, 66.5 or 84 mJ cm^−2^ for HEKas) and incubated with 1 mL of complete medium for another 2 h. Cells were collected, mixed with 0.7% low melting point agarose. The cell suspensions (40 μL) were added onto slides with 1% normal melting point agarose. The slides were soaked in lysis buffer (4 °C ) for 1 h, gently washed with PBS, immersed in electrophoresis buffer for 18 min to allow DNA denaturation, and subjected to electrophoresis for 30 min (25 V, 200 mA, Horizontal electrophoresis system, DYY-6C, Beijing Six-one Instrument plant, Beijing, China). Subsequently, the samples were neutralized with Tris–HCl (pH 7.5, 5 min × 3 times), stained with PI for 10 min, photographed by Leica fluorescence microscope, and analyzed by Comet Assay Software Project (CASP). Obvious DNA tails could be observed for HaCaTs and HEKas after UV exposure at 114 mJ cm^−2^ and 66.5 mJ cm^−2^, respectively (Additional file 1: Fig. S18). So, the corresponding UV doses were selected for the following cell protection test.

To assess the protective effect against DNA damage, HaCaTs or HEKas were pretreated with TiO_2_, ZnO, or ZIF-8 at the concentration of 60 µg mL^−1^ for 15 min and irradiated with UVB (114 mJ cm^−2^ for HaCaTs, 66.5 mJ cm^−2^ for HEKas). Comet assay was then performed as above mentioned.

*Intracellular production of ROS* HaCaT cells and HeKa cells (2.5 × 10^5^) were seeded in 24-well plates and incubated for 24 h, pretreated with TiO_2_, ZnO, or ZIF-8 at the concentration of 60 µg mL^−1^ for 15 min and irradiated with UVB or UVA (UVB: 350 mJ cm^−2^ for HaCaTs, 180 mJ cm^−2^ for HEKas; UVA: 100 mJ cm^−2^ for HaCaTs, 60 mJ cm^−2^ for HEKas). Thereafter, the cells were treated with 2′,7′—dichlorofluorescein diacetate (DCFH-DA) reactive oxygen species assay kits following the manufacture instructions. The intracellular ROS levels were assessed using confocal fluorescent microscopy and flow cytometry. (Negative control: Cells without UV exposure and without UV protection; Positive control: Cells with UV exposure but without UV protection.)

*Protective effects on mice and pig skin* The dorsal skin of each male BALB/c mouse (6-weeks old) was demarcated into six squares (1 cm × 1 cm), exposed to UV at various doses (172, 206, or 240 J m^−2^, respectively) to optimize UV dose. After three days, obvious erythema was observed for the skin with UV exposure at dose of 206 J m^−2^ and this UV dose was selected for further use (Additional file 1: Fig. S23). To optimize ZIF-8 dose, the skin squares were treated with ZIF-8 (1.5 μL) for 15 min with concentrations of 10%, 15%, or 20%, respectively, and exposed to UV at 206 J m^−2^. After three days, no obvious erythema was observed for the skin with ZIF-8 protection at the dose of 15%, so this ZIF-8 dose was selected for further use (Additional file 1: Fig. S24). To assess anti-UV effect, the skin squares were randomly treated with glycerol, TiO_2_, ZnO, or ZIF-8 (15%, 1.5 μL) for 15 min, exposed to UVB radiation (206 mJ cm^−2^) and photographed after 3 days. The mice were fed separately during the experiment. At the end time point, the skin was fixed with 4% paraformaldehyde, embedded in paraffin, sectioned with a thickness of 4 µm and subjected to Hematoxylin and Eosin (H&E) staining, Masson’s trichrome staining, or immunohistochemistry for CPD and IL-1β, respectively. The skin without protection was used as control. In these tests, the mouse and pig skin were pretreated with filters for 15 min following the World Health Organization (WHO) recommendation with minor changes, with the purpose to not affect the SPF value of these sunscreens [54, 55].

*In vivo ROS in mouse skin after UV exposure* The dorsal skin (1 cm × 1 cm) of each mouse was treated with glycerol (1.5 μL), TiO_2_, ZnO, and ZIF-8 (15%, 1.5 μL) for 15 min, respectively. Thereafter, the skin was exposed with UVB (18 mJ m^−2^) and collected after 3 h. After that, the cells were detached from the skin and stained using DCFH-DA. The ROS level was assessed using flow cytometry.

*In vivo penetration into mouse skin* The dorsal skin of the mice was demarcated into five squares (1 cm × 1 cm) after the hair was removed and randomly treated for 6 h with glycerol (1.5 μL), TiO_2_, ZnO, and ZIF-8 (15%, 1.5 μL), respectively. During the experiment, the mice were fed with 0.1 mL of water per hour by intragastric administration and heated on a pad at 37 °C. After that, the skins were topically washed with PBS (37 °C, 5 min × 3 times) and dried. The skin samples were collected and stripped for 30 times with tape, wiped with ethanol swabs for 3 times, weighed, and lysed with 70% HNO_3_ for 12 h. The levels of Zn^2+^ and Ti^4+^ in the skin were measured using ICP-MS. The skin without treatment was used as control.

*In vivo long-term toxicity* The mice were randomly divided into five groups as above mentioned. The dorsal skin was gently outlined into 2 cm × 2 cm squares using purple surgical marker and treated with glycerol (6 μL), TiO_2_, ZnO or ZIF-8 (15%, 6 μL) for 15 min every other day and 6 times in total. Three days after the last treatment, the skin, heart, liver, spleen, lung, kidney was collected, the paraffin section and the subsequent H&E staining were performed. Also, the Ti or Zn levels in heart, liver, spleen, lung, kidney, or blood were measured using ICP-MS. Additionally, blood biochemical parameters were measured to assess the system toxicity, including alkaline phosphatase (AKP), aspartate aminotransferase (AST), and alanine transaminase (ALT) for liver function, and creatinine (CRE) and serum urea nitrogen (BUN) for kidney function.

*In vivo anti-UV effect on pig* Ba-Ma miniature pig (60–90 days old, male) was anesthetized with 3.5% sodium pentobarbital (0.3 mL kg^−1^) and 10% xylazine hydrochloride injection (0.3 mL kg^−1^). During the experiment, the pig was given with supplemental anesthetics when necessary. The skin was demarcated into squares (1 cm × 1 cm) after the dorsal hair was removed. Thereafter, the skin squares were exposed to UV at various doses (442, 544, or 646 J m^−2^, respectively) to optimize UV dose. After one day, obvious erythema was induced by UV exposure at dose of 544 J m^−2^ and this UV dose was selected for further use (Additional file 1: Fig. S28). To optimize ZIF-8 dose, the skin squares were treated to ZIF-8 for 15 min with 1.5 μL of ZIF-8 at concentrations of 10%, 15%, or 20%, respectively, and exposed to UV at 544 J m^−2^. After one day, erythema was successfully inhibited by ZIF-8 protection with concentration of 15%, however, obvious erythema could still be seen for the skin with ZIF-8 treatment at 10%, so ZIF-8 dose at 15% was selected for further use (Additional file 1: Fig. S29). To assess anti-UV effect of ZIF-8 on pig, the skin squares were randomly pretreated with glycerol (1.5 μL), TiO_2_, ZnO, or ZIF-8 (15%, 1.5 μL) for 15 min, exposed to UVB radiation (544 mJ cm^−2^) and photographed again after 24 h. The skin was collected, and the paraffin section was performed, followed by H&E staining, Masson’s trichrome staining, and γH_2_AX immunofluorescence staining. The skin without protection was used as control.

*Ex vivo penetration into pig skin* Fresh pig skin was cut into pieces (1 cm × 1 cm), topically treated with glycerol, TiO_2_, ZnO or ZIF-8 (1.5 μL, 15%), respectively, and incubated at 32 °C in a humidity chamber for 6 h. The levels of Zn^2+^ and Ti^4+^ in the skins were assessed using ICP-MS after treatment following the methods in the section of “In vivo ZIF-8 penetration into mouse skin”. The skin with no treatment was used as control.

## Supplementary Information

**Additional file 1: Figure S1.** Particle size of TiO_2_, ZnO and ZIF-8. Particle size was measured using TEM and plotted thereafter. The peak sizes for TiO_2_, ZnO, and ZIF-8 were 102.1 nm, 114.8 nm and 82.3 nm, respectively. **Figure S2.** Enlarged figures for (A) Fig. 1C and (B) Fig. 1D. **Figure S3.** Characterization of ZIF-8 1:2. (A) PXRD pattern. (B) ^1^H NMR spectrum. (C) XPS spectrum. (D) N_2_ adsorption and desorption isotherms. (E) Pore size distribution. **Figure S4.** Characterization of ZIF-8 1:5. (A) PXRD pattern. (B) ^1^H NMR spectrum. (C) XPS spectrum. (D) N_2_ adsorption and desorption isotherms. (E) Pore size distribution. **Figure S5.** Characterization of ZIF-8 1:8. (A) PXRD pattern. (B) ^1^H NMR spectrum. (C) XPS spectrum. (D) N_2_ adsorption and desorption isotherms. (E) Pore size distribution. **Figure S6.** Characterization of ZIF-8 1:16. (A) PXRD pattern. (B) ^1^H NMR spectrum. (C) XPS spectrum. (D) N_2_ adsorption and desorption isotherms. (E) Pore size distribution. **Figure S7.** Physical and chemical characterizations of ZIF-8 (1:2, 1:5, 1:16). (A) TEM images of ZIF-8 (1:2, 1:5, 1:16). (B) Particle sizes of ZIF-8 (1:2, 1:5, 1:16). Particle size was measured using TEM and plotted thereafter. The sizes for ZIF-8 (1:2, 1:5, 1:16) were 164.8 ± 32.6 nm, 102.5 ± 26.8 nm, and 80.0 ± 37.7 nm, respectively. (C) Diffuse reflection spectra for ZIF-8 (1:2, 1:5, 1:16). UV reflectance, especially for UVB and UVC, was enhanced with decreasing the ratios of Zn^2+^ to 2-MeIM and reached to the highest value for ZIF-8 1:8. UV reflectance was not further increased ZIF-8 1:16. **Figure S8.** Characterization of TiO_2_. (A) PXRD pattern. (B) XPS spectrum. **Figure S9.** Characterization of ZnO. (A) PXRD pattern. (B) XPS spectrum. **Figure S10.** Characterization of MOF-5. (A) PXRD pattern. (B) ^1^H NMR spectrum. (C) XPS spectrum. (D) N_2_ adsorption and desorption isotherms. (E) Pore size distribution. **Figure S11.** Characterization of IRMOF-1. (A) PXRD pattern. (B) ^1^H NMR spectrum. (C) XPS spectrum. (D) N_2_ adsorption and desorption isotherms. (E) Pore size distribution. **Figure S12.** Characterization of Zn_3_L_3_DMF_2_. (A) PXRD pattern. (B) ^1^H NMR spectrum. (C) XPS spectrum. (D) N_2_ adsorption and desorption isotherms. (E) Pore size distribution. **Figure S13.** Physical and chemical characterizations of zinc-based MOFs. (A-C) TEM images. (A) MOF-5. (B) IRMOF-1. (C) Zn_3_L_3_DMF_2_. (D-F) Particle size distributions of MOF-5, IRMOF-1 and Zn_3_L_3_DMF_2_. Particle size was measured using TEM and plotted thereafter. The sizes were 310.6 ± 142.4 nm, 47.1 ± 13.6 nm, and 256.0 ± 91.3 nm for MOF-5, IRMOF-1, and Zn_3_L_3_DMF_2_, respectively. (G) The zeta potentials of TiO_2_, ZnO, ZIF-8, MOF-5, IRMOF-1, and Zn_3_L_3_DMF_2_ were 31.7 ± 0.6 mV, 16.4 ± 0.7 mV, 29.5 ± 0.8 mV, − 9.9 ± 1.5 mV, − 7.0 ± 0.6 mV, and − 5.6 ± 0.6 mV, respectively. (H) The degradation of zinc-based MOFs in artificial sweat (pH 6.5, 32 °C). ZIF-8 (0.5 mg) exhibited the lowest degradation rate relative to that of MOF-5 (0.5 mg), IRMOF-1 (0.5 mg), and Zn_3_L_3_DMF_2_ (0.5 mg). **Figure S14.** EPR spectra of POBN-OH spin abduct signal produced by TiO_2_ suspensions in ethanol. (A) TiO_2_ at 800 µg mL^−1^, (B) TiO_2_ at 50 µg mL^−1^. No obvious EPR signal was detected for TiO_2_ at 50 µg mL^−1^. **Figure S15.** EPR spectra of POBN-OH spin abduct signal produced by suspensions of TiO_2_, ZnO, and Zn-based MOFs at 800 µg mL^−1^ in ethanol. (A) TiO_2_, (B) ZnO, (C) ZIF-8, (D) MOF-5, (E) IRMOF-1, (F) Zn_3_L_3_DMF_2_. ZnO produced most free radical of ·OH (1.3 × 10^12^ spins/mm^3^), followed by TiO_2_ (5.9 × 10^11^ spins/mm^3^), MOF-5 (4.1 × 10^11^ spins/mm^3^), Zn_3_L_3_DMF_2_ (2.1 × 10^11^ spins/mm^3^), IRMOF-1 (6.2 × 10^10^ spins/mm^3^) and ZIF-8 (2.3 × 10^10^ spins/mm^3^), suggesting Zn-based MOFs induced much less EPR signal compared to TiO_2_ and ZnO after UV exposure. **Figure S16.** UV absorbance of ZIF-8, TiO_2_, ZnO and 2-MeIM. ZIF-8 showed a higher UV absorbance compared to ZnO. Also, ZIF-8 revealed a higher UVB absorption relative to TiO_2_, though UVA absorption of is lower. **Figure S17.** Cell viability of (A) HaCaTs or (B) HEKas after exposed with UV in various doses. **Figure S18.** Fluorescence images of DNA tail after (A) HaCaTs or (B) HEKas were exposed to UV in various doses. **Figure S19.** ROS levels in HaCaTs after UVB exposure. (A) Confocal fluorescent images (Blue, nucleus; Green, ROS positive.). (B) Flow cytometry analyses of free radical levels in HaCaTs with/without protections. HaCaTs with ZIF-8 pretreatment showed no obvious increase of ROS. However, ROS were elevated for the cells without protection or with the protections of TiO_2_, ZnO, MOF-5, IRMOF-1 or Zn_3_L_3_DMF_2_. **Figure S20.** ROS levels in HEKas after UVB exposure. (A) Confocal fluorescence images (Blue, nucleus; Green, ROS positive.) and (B) flow cytometry analyses of free radicals in HEKas with/without protections. HaCaTs with ZIF-8 pretreatment showed less ROS production relative to that for the cells without protection or with the protections of TiO_2_, ZnO, MOF-5, IRMOF-1 or Zn_3_L_3_DMF_2_. **Figure S21.** ROS levels in HaCats after UVA exposure. (A) Confocal fluorescent images (Blue, nucleus; Green, ROS). (B) Flow cytometry analyses of free radical level in HaCats with/without protections. HaCaTs with ZIF-8 pretreatment showed no obvious ROS production. More ROS production were observed in the cells without protection or with the protections of TiO_2_, ZnO, MOF-5, IRMOF-1 or Zn_3_L_3_DMF_2_. **Figure S22.** ROS measurement in HEKas after UVA exposure. (A) Confocal fluorescent images (Blue, nucleus; Green, ROS.). (B) Flow cytometry analyses of free radicals in HEKas with/without protections. HEKas with ZIF-8 protection showed less ROS production relative to that for the groups of No protection, IRMOF-1, TiO_2_, ZnO, MOF-5, or Zn_3_L_3_DMF_2_. **Figure S23.** UV dose optimization on mouse skin. Images of mouse dorsal skin three days after UV exposure at different doses. UV dose of 206 J m^−2^ was selected for further use because erythema was observed at this dose. **Figure S24.** ZIF-8 dose optimization. Images of mouse skin three days after UV exposure with protections of TiO_2_, ZnO, and ZIF-8 at different doses. The dose of 15% was selected for further in vivo mouse study, because some sunburn was observed for TiO_2_ and ZnO mice at this dose, while no obvious erythema was observed for ZIF-8 mice at dose of 15%. **Figure S25.** Digital graphs of mouse dorsal skin three days after UV exposure with the protections of TiO_2_, ZnO, or ZIF-8. ZIF-8 group showed less ulceration, edema or erythema compared to no protection or glycerol group. **Figure S26.** ROS in mouse skin after UV exposure. (A) Quantitative and (B) qualitative analyses of free radical level in the skin with/without protections. **Figure S27.** Microscope photographs of the mouse skin with (A) Masson’s Trichrome staining or (B) IL-1β immunohistochemistry. UV disturbed collagen distribution and decreased density for no protection and glycerol groups, while ZIF-8 group showed a normal collagen appearance. ZIF-8 also inhibited the skin expression of IL-1β after UV exposure. **Figure S28.** UV dose optimization on pig skin. Digital images of pig dorsal skin 24 h after UV exposure. UV at 544 J m^−2^ could induce obvious erythema. **Figure S29.** ZIF-8 dose optimization against UV exposure on pig skin. Digital graphs of pig dorsal skin 24 h after UV exposure with protections of ZIF-8 at different doses. ZIF-8 at 15% obviously inhibited erythema formation. **Figure S30.** Digital graphs of pig dorsal skin 24 h after UV exposure with the protections of TiO_2_, ZnO, or ZIF-8. ZIF-8 obviously inhibited erythema formation. **Figure S31.** Blood biochemical analyses after mice were treated with TiO_2_, ZnO, or ZIF-8 for six times in 15 days. (A-C) Serum levels of (A) AKP, (B) ALT, and (C) AST for liver function analyses. (D-E) Serum levels of (D) BUN, and (E) CRE for kidney function analyses. Both liver and kidney functions were not affected by TiO_2_, ZnO, or ZIF-8. **Figure S32.** H&E images of main tissues after mice were treated with TiO_2_, ZnO, or ZIF-8 for six times in 15 days. No obvious tissue damage was observed for heart, liver, spleen, lung, and kidney in all these groups. **Figure S33.** The accumulations of Ti or Zn in blood and tissues after TiO_2_, ZnO, or ZIF-8 were applied for six times in 15 days. (A, B) Ti levels in (A) heart, liver, spleen, lung, and kidney and (B) blood for the mice with TiO_2_ treatment. (C, D) Zn levels in (C) heart, liver, spleen, lung, and kidney and (D) blood for the mice with ZnO or ZIF-8 treatments.

## Abbreviations

MOFs: Metal–organic frameworks
ROS: Reactive oxygen species
Egs: Bandgap energies
SPF: Sun protection factor
RT: Room temperature
ZnO: Zinc oxide
TiO_2_: Titanium oxide
Zn(Ac)_2_·2H_2_O: Zinc acetate dihydrate
H_2_BDC: Terephthalic acid
DMF: N,N-dimethylformamide
2-MeIM: 2-Methylimidazole
DMSO-*d*_*6*_: Dimethyl sulfoxide-*d*_*6*_
LH_2_: 4,4’-Stilbenedicarboxylic acid
DMEM: Dulbecco’s modified eagle medium
MEM: Minimum essential medium
FBS: Fetal bovine serum
MTT: Thiazolyl blue
γ-H_2_AX: Phospho-histone H_2_AX (Ser139) antibody
DCFH-DA: 2,7-Dichlorofluorescein diacetate
HaCaTs: Human immortalized epidermal keratinocytes
HEKas: Human epithelial keratinocytes
PXRD: Powder X-ray diffraction
TEM: Transmission electron microscope
ICP-MS: Inductively coupled plasma mass spectrometry
XPS: X-ray photoelectron spectroscopy
TGA: Thermogravimetric analysis
EPR: Electron paramagnetic resonance
POBN: α-(4-Pyridyl N-oxide)-N-tert-butylnitrone
PI: Propidium iodide
H&E: Hematoxylin and Eosin
