# Supplementary material for: Zinc-metal–organic frameworks with tunable UV diffuse-reflectance as sunscreens
Source: J Nanobiotechnology. 2022 Feb 19;20:87. doi: 10.1186/s12951-022-01292-1 (PMC8858458; doi:10.1186/s12951-022-01292-1)
Supplement: Supplementary file 1 — Additional file 1: Figure S1. Particle size of TiO2, ZnO and ZIF-8. Particle size was measured using TEM and plotted thereafter. The peak sizes for TiO2, ZnO, and ZIF-8 were 102.1 nm, 114.8 nm and 82.3 nm, respectively. Figure S2. Enlarged figures for (A) Fig. 1C and (B) Fig. 1D. Figure S3. Characterization of ZIF-8 1:2. (A) PXRD pattern. (B) 1H NMR spectrum. (C) XPS spectrum. (D) N2 adsorption and desorption isotherms. (E) Pore size distribution. Figure S4. Characterization of ZIF-8 1:5. (A) PXRD pattern. (B) 1H NMR spectrum. (C) XPS spectrum. (D) N2 adsorption and desorption isotherms. (E) Pore size distribution. Figure S5. Characterization of ZIF-8 1:8. (A) PXRD pattern. (B) 1H NMR spectrum. (C) XPS spectrum. (D) N2 adsorption and desorption isotherms. (E) Pore size distribution. Figure S6. Characterization of ZIF-8 1:16. (A) PXRD pattern. (B) 1H NMR spectrum. (C) XPS spectrum. (D) N2 adsorption and desorption isotherms. (E) Pore size distribution. Figure S7. Physical and chemical characterizations of ZIF-8 (1:2, 1:5, 1:16). (A) TEM images of ZIF-8 (1:2, 1:5, 1:16). (B) Particle sizes of ZIF-8 (1:2, 1:5, 1:16). Particle size was measured using TEM and plotted thereafter. The sizes for ZIF-8 (1:2, 1:5, 1:16) were 164.8 ± 32.6 nm, 102.5 ± 26.8 nm, and 80.0 ± 37.7 nm, respectively. (C) Diffuse reflection spectra for ZIF-8 (1:2, 1:5, 1:16). UV reflectance, especially for UVB and UVC, was enhanced with decreasing the ratios of Zn2+ to 2-MeIM and reached to the highest value for ZIF-8 1:8. UV reflectance was not further increased ZIF-8 1:16. Figure S8. Characterization of TiO2. (A) PXRD pattern. (B) XPS spectrum. Figure S9. Characterization of ZnO. (A) PXRD pattern. (B) XPS spectrum. Figure S10. Characterization of MOF-5. (A) PXRD pattern. (B) 1H NMR spectrum. (C) XPS spectrum. (D) N2 adsorption and desorption isotherms. (E) Pore size distribution. Figure S11. Characterization of IRMOF-1. (A) PXRD pattern. (B) 1H NMR spectrum. (C) XPS spectrum. (D) N2 adsorption and de [file 12951_2022_1292_MOESM1_ESM.docx]

Additional Information

Zinc-Metal-Organic Frameworks with tunable UV diffuse-reflectance as sunscreens

Jisheng Xiao^a,b,c,1,*^ Haishan Li,^c,1^ Wanling Zhao,^c^ Chengyuan Cai, ^b^ Tingting You, ^b^ Zhenyu Wang, ^b^ Mengling Wang,^d^ Feng Zeng,^e^ Jinmei Cheng,^b^ Jiaxin Li,^b^ Xiaopin Duan^b*^

Dr. Jisheng Xiao,

^a^Translational Medicine Research Center, Zhujiang Hospital, Southern Medical University/The Second School of Clinical Medicine, Southern Medical University, Guangzhou, Guangdong, 510515, China.

^b^Cancer Research Institute, School of Basic Medical Sciences, Southern Medical University, 510515, Guangzhou, China.

^c^Science and Technology Innovation Center, Guangzhou University of Chinese Medicine, 510405, Guangzhou, China.

E-mail: jsxiao2021@163.com, jsxiao@gzucm.edu.cn.

Dr. Xiaopin Duan, Chengyuan Cai, Tingting You, Zhenyu Wang, Jinmei Cheng, Jiaxin Li,
^b^Cancer Research Institute, School of Basic Medical Sciences, Southern Medical University, 510515, Guangzhou, China.

E-mail: xduan85@i.smu.edu.cn

Haishan Li, Wanling Zhao,
^c^Department of Pharmacology, School of Pharmaceutical Sciences, Guangzhou University of Chinese Medicine, 510006, Guangzhou, China.

Dr. Mengling Wang,

^d^Department of Pharmacology, School of Pharmacy, Fudan University, Minhang Hospital, Shanghai 201203, China.

Dr. Feng Zeng,

^e^Artemisinin Research Center, Institute of Science and Technology, The First Affiliated Hospital, The First Clinical Medical School, Lingnan Medical Research Center, Guangzhou University of Chinese Medicine, Guangzhou, 510405, China

^1^These authors contribute equally to this work.

^*^Corresponding authors.

**Additional equations:**

$$ᾱ=-\frac{8}{\pi^{5}}\left[ 4\pi\left[ \frac{\pi^{4}}{16}-I_{1}I_{2} \right]+\left( 4I_{4}-2I_{5} \right)\frac{\pi^{2}}{4}+ I_{1}\left( 2I_{3}-\pi^{2}\alpha_{0} \right) \right] (S2.1)$$

$$\alpha_{n}=\frac{(n+1)(\epsilon-1)}{\epsilon_{1}(n\epsilon+n+1)} (S2.2)$$

with

ϵ_1_: the dielectric constants of the semiconductor material;

ϵ_2_: the dielectric constants of the surrounding medium;

ϵ= ϵ_1_/ ϵ_2_

$$I_{1}= \int_{0}^{\pi} dx_{e}\int_{0}^{\pi} dx_{h}{sin}^{2}x_{e}{sin}^{2}x_{h}\mathcal{R}_{0}\left( x_{e},x_{h} \right) (S2.3)$$

$$I_{2}= \int_{0}^{\pi} dx_{e}\int_{0}^{\pi} dx_{h}\frac{1}{x_{>}}{sin}^{2}x_{e}{sin}^{2}x_{h} (S2.4)$$

$$I_{3}= \pi\int_{0}^{\pi} dx{sin}^{2}x\sum_{n=0}^{\infty} \alpha_{n}[\frac{x}{\pi}]^{2n} (S2.5)$$

$$I_{4}= \int_{0}^{\pi} dx_{e}\int_{0}^{\pi} dx_{h}{sin}^{2}x_{e}{sin}^{2}x_{h} \times\sum_{n=0}^{n} \alpha_{n}[\frac{x_{e}x_{h}}{\pi^{2}}]^{n}\frac{\mathcal{R}_{n}\left( x_{e},x_{h} \right)}{2n+1} (S2.6)$$

$$I_{5}= \int_{0}^{\pi} dx_{e}\int_{0}^{\pi} dx_{h}{sin}^{2}x_{e}{sin}^{2}x_{h}\mathcal{R}_{0}\left( x_{e},x_{h} \right) \times\sum_{n=0}^{n} \alpha_{n}[[\frac{x_{e}}{\pi}]^{2n}+ \left[ \frac{x_{e}}{\pi}]^{2n} \right] (S2.7)$$

**Additional figures**

**Figure S1.** Particle size of TiO_2_, ZnO and ZIF-8. Particle size was measured using TEM and plotted thereafter. The peak sizes for TiO_2_, ZnO, and ZIF-8 were 102.1 nm,114.8 nm and 82.3 nm, respectively.

**Figure S2.** Enlarged figures for (A) Figure 1C and (B) Figure 1D.

**Figure S3.** Characterization of ZIF-8 1:2. (A) PXRD pattern. (B) ^1^H NMR spectrum. (C) XPS spectrum. (D) N_2_ adsorption and desorption isotherms. (E) Pore size distribution.

**Figure S4.** Characterization of ZIF-8 1:5. (A) PXRD pattern. (B) ^1^H NMR spectrum. (C) XPS spectrum. (D) N_2_ adsorption and desorption isotherms. (E) Pore size distribution.

**Figure S5.** Characterization of ZIF-8 1:8. (A) PXRD pattern. (B) ^1^H NMR spectrum. (C) XPS spectrum. (D) N_2_ adsorption and desorption isotherms. (E) Pore size distribution.

**Figure S6.** Characterization of ZIF-8 1:16. (A) PXRD pattern. (B) ^1^H NMR spectrum. (C) XPS spectrum. (D) N_2_ adsorption and desorption isotherms. (E) Pore size distribution.

**Figure S7.** Physical and chemical characterizations of ZIF-8 (1:2, 1:5, 1:16). (A) TEM images of ZIF-8 (1:2, 1:5, 1:16). (B) Particle sizes of ZIF-8 (1:2, 1:5, 1:16). Particle size was measured using TEM and plotted thereafter. The sizes for ZIF-8 (1:2, 1:5, 1:16) were 164.8 ± 32.6 nm, 102.5 ± 26.8 nm, and 80.0 ± 37.7 nm, respectively. (C) Diffuse reflection spectra for ZIF-8 (1:2, 1:5, 1:16). UV reflectance, especially for UVB and UVC, was enhanced with decreasing the ratios of Zn^2+^ to 2-MeIM and reached to the highest value for ZIF-8 1:8. UV reflectance was not further increased ZIF-8 1:16.

**Figure S8.** Characterization of TiO_2_. (A) PXRD pattern. (B) XPS spectrum.

**Figure S9.** Characterization of ZnO. (A) PXRD pattern. (B) XPS spectrum.

**Figure S10.** Characterization of MOF-5. (A) PXRD pattern. (B) ^1^H NMR spectrum. (C) XPS spectrum. (D) N_2_ adsorption and desorption isotherms. (E) Pore size distribution.

**Figure S11.** Characterization of IRMOF-1. (A) PXRD pattern. (B) ^1^H NMR spectrum. (C) XPS spectrum. (D) N_2_ adsorption and desorption isotherms. (E) Pore size distribution.

**Figure S12.** Characterization of Zn_3_L_3_DMF_2_. (A) PXRD pattern. (B) ^1^H NMR spectrum. (C) XPS spectrum. (D) N_2_ adsorption and desorption isotherms. (E) Pore size distribution.

**Figure S13.** Physical and chemical characterizations of zinc-based MOFs. (A-C) TEM images. (A) MOF-5. (B) IRMOF-1. (C) Zn_3_L_3_DMF_2_. (D-F) Particle size distributions of MOF-5, IRMOF-1 and Zn_3_L_3_DMF_2_. Particle size was measured using TEM and plotted thereafter. The sizes were 310.6 ± 142.4 nm, 47.1 ± 13.6 nm, and 256.0 ± 91.3 nm for MOF-5, IRMOF-1, and Zn_3_L_3_DMF_2_, respectively. (G) The zeta potentials of TiO_2_, ZnO, ZIF-8, MOF-5, IRMOF-1, and Zn_3_L_3_DMF_2_ were 31.7 ± 0.6 mV, 16.4 ± 0.7 mV, 29.5 ± 0.8 mV, - 9.9 ± 1.5 mV, - 7.0 ± 0.6 mV, and - 5.6 ± 0.6 mV, respectively. (H) The degradation of zinc-based MOFs in artificial sweat (pH 6.5, 32 ˚C). ZIF-8 (0.5 mg) exhibited the lowest degradation rate relative to that of MOF-5 (0.5 mg), IRMOF-1 (0.5 mg), and Zn_3_L_3_DMF_2_ (0.5 mg).

**Figure S14.** EPR spectra of POBN-OH spin abduct signal produced by TiO_2_ suspensions in ethanol. (A) TiO_2_ at 800 µg mL^-1^, (B) TiO_2_ at 50 µg mL^-1^. No obvious EPR signal was detected for TiO_2_ at 50 µg mL^-1^.

**Figure S15.** EPR spectra of POBN-OH spin abduct signal produced by suspensions of TiO_2_, ZnO, and Zn-based MOFs at 800 µg mL^-1^ in ethanol. (A) TiO_2_, (B) ZnO, (C) ZIF-8, (D) MOF-5, (E) IRMOF-1, (F) Zn_3_L_3_DMF_2_. ZnO produced most free radical of ·OH (1.3 × 10^12^ spins/mm^3^), followed by TiO_2_ (5.9 × 10^11^ spins/mm^3^), MOF-5 (4.1×10^11^ spins/mm^3^), Zn_3_L_3_DMF_2_ (2.1 × 10^11^ spins/mm^3^), IRMOF-1 (6.2 × 10^10^ spins/mm^3^) and ZIF-8 (2.3 × 10^10^ spins/mm^3^), suggesting Zn-based MOFs induced much less EPR signal compared to TiO_2_ and ZnO after UV exposure.

**Figure S16.** UV absorbance of ZIF-8, TiO_2_, ZnO and 2-MeIM. ZIF-8 showed a higher UV absorbance compared to ZnO. Also, ZIF-8 revealed a higher UVB absorption relative to TiO_2_, though UVA absorption of is lower.

**Figure S17.** Cell viability of (A) HaCaTs or (B) HEKas after exposed with UV in various doses.

**Figure S18.** Fluorescence images of DNA tail after (A) HaCaTs or (B) HEKas were exposed to UV in various doses.

**Figure S19.** ROS levels in HaCaTs after UVB exposure. (A) Confocal fluorescent images (Blue, nucleus; Green, ROS positive.). (B) Flow cytometry analyses of free radical levels in HaCaTs with/without protections. HaCaTs with ZIF-8 pretreatment showed no obvious increase of ROS. However, ROS were elevated for the cells without protection or with the protections of TiO_2_, ZnO, MOF-5, IRMOF-1 or Zn_3_L_3_DMF_2_.

**Figure S20.** ROS levels in HEKas after UVB exposure. (A) Confocal fluorescence images (Blue, nucleus; Green, ROS positive.) and (B) flow cytometry analyses of free radicals in HEKas with/without protections. HaCaTs with ZIF-8 pretreatment showed less ROS production relative to that for the cells without protection or with the protections of TiO_2_, ZnO, MOF-5, IRMOF-1 or Zn_3_L_3_DMF_2_.

**Figure S21.** ROS levels in HaCats after UVA exposure. (A) Confocal fluorescent images (Blue, nucleus; Green, ROS). (B) Flow cytometry analyses of free radical level in HaCats with/without protections. HaCaTs with ZIF-8 pretreatment showed no obvious ROS production. More ROS production were observed in the cells without protection or with the protections of TiO_2_, ZnO, MOF-5, IRMOF-1 or Zn_3_L_3_DMF_2_.

**Figure S22.** ROS measurement in HEKas after UVA exposure. (A) Confocal fluorescent images (Blue, nucleus; Green, ROS.). (B) Flow cytometry analyses of free radicals in HEKas with/without protections. HEKas with ZIF-8 protection showed less ROS production relative to that for the groups of No protection, IRMOF-1, TiO_2_, ZnO, MOF-5, or Zn_3_L_3_DMF_2_.

**Figure S23.** UV dose optimization on mouse skin. Images of mouse dorsal skin three days after UV exposure at different doses. UV dose of 206 J m^-2^ was selected for further use because erythema was observed at this dose.

**Figure S24.** ZIF-8 dose optimization. Images of mouse skin three days after UV exposure with protections of TiO_2_, ZnO, and ZIF-8 at different doses. The dose of 15 % was selected for further *in vivo* mouse study, because some sunburn was observed for TiO_2_ and ZnO mice at this dose, while no obvious erythema was observed for ZIF-8 mice at dose of 15 %.

**Figure S25.** Digital graphs of mouse dorsal skin three days after UV exposure with the protections of TiO_2_, ZnO, or ZIF-8. ZIF-8 group showed less ulceration, edema or erythema compared to no protection or glycerol group.

**Figure S26.** ROS in mouse skin after UV exposure. (A) Quantitative and (B) qualitative analyses of free radical level in the skin with/without protections.

**Figure S27.** Microscope photographs of the mouse skin with (A) Masson’s Trichrome staining or (B) IL-1β immunohistochemistry. UV disturbed collagen distribution and decreased density for no protection and glycerol groups, while ZIF-8 group showed a normal collagen appearance. ZIF-8 also inhibited the skin expression of IL-1β after UV exposure.

**Figure S28.** UV dose optimization on pig skin. Digital images of pig dorsal skin 24 hrs after UV exposure. UV at 544 J m^-2^ could induce obvious erythema.

**Figure S29.** ZIF-8 dose optimization against UV exposure on pig skin. Digital graphs of pig dorsal skin 24 hrs after UV exposure with protections of ZIF-8 at different doses. ZIF-8 at 15% obviously inhibited erythema formation.

**Figure S30.** Digital graphs of pig dorsal skin 24 hrs after UV exposure with the protections of TiO_2_, ZnO, or ZIF-8. ZIF-8 obviously inhibited erythema formation.

**Figure S31.** Blood biochemical analyses after mice were treated with TiO_2_, ZnO, or ZIF-8 for six times in 15 days. (A-C) Serum levels of (A) AKP, (B) ALT, and (C) AST for liver function analyses. (D-E) Serum levels of (D) BUN, and (E) CRE for kidney function analyses. Both liver and kidney functions were not affected by TiO_2_, ZnO, or ZIF-8.

**Figure S32.** H&E images of main tissues after mice were treated with TiO_2_, ZnO, or ZIF-8 for six times in 15 days. No obvious tissue damage was observed for heart, liver, spleen, lung, and kidney in all these groups.

**Figure S33.** The accumulations of Ti or Zn in blood and tissues after TiO_2_, ZnO, or ZIF-8 were applied for six times in 15 days. (A, B) Ti levels in (A) heart, liver, spleen, lung, and kidney and (B) blood for the mice with TiO_2_ treatment. (C, D) Zn levels in (C) heart, liver, spleen, lung, and kidney and (D) blood for the mice with ZnO or ZIF-8 treatments.
